# Microbial Community Imbalance Drives Nitrous Oxide Emissions from Strongly Acidic Soil—Insights from a Laboratory Experiment with Microbial Inhibitors

**DOI:** 10.3390/biology14060621

**Published:** 2025-05-28

**Authors:** Waqar Ahmed, Hongyang Gong, Xiaoxiao Xiang, Runze Chen, Yumeng Xu, Wenxuan Shi, Binzhe Li, Junhui Yin, Qing Chen

**Affiliations:** 1 State Key Lab of Biocontrol, Guangdong Provincial Key Laboratory of Plant Stress Biology, School of Agriculture and Biotechnology, Shenzhen Campus of Sun Yat-sen University, Shenzhen 518107, China; waqarahmedupr@gmail.com (W.A.); hygong@cau.edu.cn (H.G.); 2 College of Resources and Environmental Sciences, China Agricultural University, Beijing 100193, China; xiangxx@cau.edu.cn (X.X.); crz15751136686@163.com (R.C.); 13470700223@163.com (Y.X.); qchen@cau.edu.cn (Q.C.); 3 Teagasc, Environmental Research Centre, Johnstown Castle, Y35 TC97 Co. Wexford, Ireland; wenxuan.shi@teagasc.ie; 4 College of Engineering (Key Laboratory for Clean Renewable Energy Utilization Technology, Ministry of Agriculture), China Agricultural University, Beijing 100083, China; libinzhe@cau.edu.cn

**Keywords:** nitrous oxide, microbial inhibition, nitrogen dynamics, dissolved organic carbon, acidic soil

## Abstract

Nitrous oxide (N_2_O) is a potent greenhouse gas with intensive emissions from acidic soil. This study explored the impact of the disruption of the microbial balance from microbial inhibitors (streptomycin and cycloheximide) on soil’s N_2_O emission and nitrogen (N) dynamics. Under all the conditions examined, biotic processes accounted for 96–98% of total N_2_O emissions. High concentrations of streptomycin (6 and 10 mg g^−1^) reduced N_2_O emissions from 2.24 μg kg^−1^ h^−1^ to 1.93 μg kg^−1^ h^−1^ and 2.12 μg kg^−1^ h^−1^, respectively, whereas lower concentrations (2 and 4.5 mg g^−1^) increased emissions from 2.24 μg kg^−1^ h^−1^ to 2.95 μg kg^−1^ h^−1^ and 3.27 μg kg^−1^ h^−1^, respectively. Lower cycloheximide (2 and 4.5 mg g^−1^) significantly enhanced N_2_O emissions, reaching 9.15 μg kg^−1^ h^−1^ and 5.68 μg kg^−1^ h^−1^, respectively, whereas higher dosages (6 mg g^−1^ and 10 mg g^−1^) inhibited N_2_O emissions, reducing them to 5.55 μg kg^−1^ h^−1^ and 4.84 μg kg^−1^ h^−1^, respectively. Carbon dioxide (CO_2_) emissions generally decreased with increasing inhibitor dosages but significantly increased at 2 mg g^−1^ and 4.5 mg g^−1^ streptomycin. The inhibitors also altered soil N and carbon (C) dynamics, increasing ammonium (NH_4_^+^-N), dissolved organic nitrogen (DON), and dissolved organic carbon (DOC) levels. Pearson correlation analysis indicated that N_2_O emission was negatively correlated with cycloheximide dosage (R = −0.68, *p* < 0.001), NH_4_^+^-N (R = −0.31, *p* < 0.001) and DOC content (R = −0.57, *p* < 0.05). These findings highlight the consequences of microbial disruption on N_2_O emission and the complex microbial interactions in acidic soils. High concentrations of microbial inhibitors effectively reduce N_2_O emissions by suppressing key microbial groups in nitrification and denitrification. Conversely, lower concentrations may prompt compensatory responses from surviving microorganisms, resulting in increased N_2_O production. Future research should focus on sustainable management strategies to mitigate N_2_O emissions while preserving the soil’s microbial community.

## Simple Summary

This study examined how adding different amounts of two microbial inhibitors (streptomycin and cycloheximide) affects nitrous oxide (N_2_O) emissions in strongly acidic soil. High concentrations of streptomycin (6 and 10 mg g^−1^) reduced N_2_O emissions, whereas lower concentrations (2 and 4.5 mg g^−1^) increased emissions. Cycloheximide initially elevated N_2_O emissions compared to the control, but emissions decreased as the concentration of cycloheximide increased. The inhibitors also altered soil nitrogen and carbon levels. This study highlights that microbial inhibitors can disrupt soil microbial communities, leading to changes in N_2_O emissions. High concentrations of inhibitors can reduce emissions by suppressing certain microbes, but lower concentrations may cause surviving microbes to produce more N_2_O.

## 1. Introduction

The mitigation of greenhouse gas (GHG) emissions is one of the most pressing global challenges [1]. Nitrous oxide (N_2_O) is the third most significant contributor to global warming and a major ozone-depleting substance [2,3]. Agricultural soils are the primary source of anthropogenic N_2_O emissions, accounting for approximately 60% of global emissions [4]. Soil pH is the major factor affecting soil N_2_O emission, with some studies showing that acidic soil is an important emission source of N_2_O [5]. It has been reported that acidic soil (pH 5.0) emitted three times more N_2_O than alkaline soil (pH 8.0) with a similar application of nitrogen (N) fertilizer [6]. Soil acidification is an increasing issue as more than half of worldwide arable land is becoming acidic [7]. Understanding the mechanisms driving N_2_O emission in acidic soils is of paramount importance for developing effective N_2_O emission mitigation strategies.

N_2_O emission in acidic soils is driven by a combination of biological and abiotic pathways [8]. Chemical processes, such as chemodenitrification, where reactive N intermediates (e.g., HNO_2_) decompose abiotically under low pH conditions, have been identified as significant contributors to N_2_O emission in acidic environments [9]. For example, research in tropical soils has attributed over 50% of N_2_O fluxes to abiotic reactions involving Fe^2+^-mediated nitrite reduction [10,11]. However, emerging evidence highlights the resilience and adaptability of acid-tolerant microbial communities in driving N_2_O production [12]. Prokaryotic processes, including bacterial nitrification, denitrification, and nitrifier denitrification, remain active even at pH < 4.5, facilitated by acidophilic taxa such as *Acidithiobacillus* and *Acidobacteriota* [13]. Eukaryotic contributions, particularly fungal denitrification, are increasingly recognized as critical, as fungi exhibit greater tolerance to acidic conditions than many bacteria [14]. However, the interplay between prokaryotic and eukaryotic communities in regulating N_2_O flux, and how their functional redundancy or competition modulates overall emissions, remains unresolved. This knowledge gap hinders the development of microbial-focused mitigation strategies.

Soil microorganisms coexist in a dynamic equilibrium, each contributing to nutrient cycling and soil structure maintenance [15]. Agricultural management practices, such as liming, the application of organic amendments, and pesticide use, strongly disrupt the balance of microbial populations, thus influencing N_2_O emissions [16,17,18]. For instance, an increased pH decreases the contribution of fungi to N_2_O but increases bacterial N_2_O production in acidic soil [19]. The addition of manure enhances N availability and organic C, resulting in stimulating nitrification and denitrification processes dominated by prokaryotes (e.g., *Nitrosomonas* and *Pseudomonas*) [20]. Meanwhile, the application of bactericide and fungicide influences N cycling; the effects depend on soil type and conditions [21]. Disease control measures, especially the application of pesticides, indirectly influence the interaction between prokaryotes and eukaryotes, highlighting the need to disentangle these microbial networks to refine N_2_O mitigation strategies [22]. However, inhibitor effects are dose-dependent and may inadvertently stimulate non-target microbial groups or modify substrate availability, complicating their interpretation. Disentangling the roles of prokaryotes and eukaryotes in acidic soils is essential to clarify their individual and interactive effects on N_2_O dynamics, particularly under scenarios of ecological imbalance induced by agricultural interventions.

The objectives of this study were (i) to distinguish the contribution of biological and abiotic processes to N_2_O emissions in acidic soils; (ii) to examine the N_2_O emissions response to the inhibition of prokaryotic and eukaryotic microorganisms; iii) and to explore the relationship of N_2_O emissions to the dosage of prokaryotic and eukaryotic inhibitors. A microcosm experiment was conducted using acidic vegetable soil treated with gradient concentrations of cycloheximide (a eukaryotic inhibitor) and streptomycin (a prokaryotic inhibitor), both individually and in combination. Gas (N_2_O and CO_2_) fluxes, soil physicochemical properties, and substrate dynamics were monitored to evaluate dose–response relationships. By elucidating the roles of prokaryotic–eukaryotic interactions and inhibitor dosage in regulating N_2_O emission, this work advances a mechanistic understanding of N cycling in acidic agroecosystems and informs sustainable management practices to mitigate greenhouse gas emission.

## 2. Materials and Methods

### 2.1. Soil Sampling

The soil used was collected from Qunfeng Town, Zhuzhou City, Hunan Province 27°43′57″ N, 113°4′0″ E. This region’s mean annual temperature is 18 °C, and the mean annual precipitation is 1200–1600 mm. Soil samples of the 0–20 cm soil layer were collected in April 2019. The sampled soil was sieved through a 2 mm mesh and air-dried. This study’s reported measurements and calculations were based on oven-dry soil mass. The soil had a pH of 3.9, with a texture composed of 10.8% clay, 57.3% silt, and 31.9% sand, and it contained 1.94 g kg^−1^ of total nitrogen (TN), 18.7 g kg^−1^ of soil organic C (SOM), a C/N ratio of 9.66, 86.5 mg kg^−1^ of available phosphorus (Olsen-P), and 92 mg kg^−1^ of exchangeable potassium (AK).

### 2.2. Experimental Design

To quantitatively assess the relative contributions of biotic and abiotic processes to soil N_2_O emissions, the soil samples were divided into two distinct groups: sterilized and non-sterilized treatments. Both groups were subjected to three N amendment conditions: (i) control (CK); (ii) ammonium sulfate (NH_4_)_2_SO_4_ application at 100 mg N kg^−1^ soil (NH_4_^+^-N); and (iii) potassium nitrate (KNO_3_) application at 100 mg N kg^−1^ soil (NO_3_^−^-N). (NH_4_)_2_SO_4_ and KNO_3_ were procured from Sinopharm Chemical Reagent Co., Ltd. (Shanghai, China). Each treatment had three replicates. For sterilized incubation, soil samples were sterilized by ^60^Co-γ irradiation with a dose of 25 kGy for 72 h, which is a widely used and effective method to sterilize soil samples [23]. Sterilization of media and other solutions was achieved by autoclaving them for 30 min at 121 °C. Twenty grams of air-dried soil was pre-incubated in 120 mL serum flasks at 25 °C and 40% water holding capacity (WHC) for 7 d. The soil water holding capacity was 28% *w*/*w* water, A pipette was used to dropwise add deionized water with dissolved (NH_4_)_2_SO_4_ or KNO_3_ to the bottles, and a syringe was used to spray deionized water evenly onto the soil surface so that the soil moisture reached 70% of WHC. The bottles were then sealed with a rubber stopper and cultured in a shading incubator at 25 °C.

The microbial inhibition experiment was designed to investigate the effects of prokaryotic and eukaryotic inhibitors on soil N_2_O emission using streptomycin and cycloheximide (Sigma-Aldrich Corporation, St. Louis, MO, USA) individually. The experimental design consisted of 25 treatments, each with three replicates, including (1) a control group (CK) without inhibitors (0 mg g^−^^1^ streptomycin, 0 mg g^−^^1^ cycloheximide); (2) 4 streptomycin-only treatments (ST) at concentrations of 2, 4.5, 6, and 10 mg g^−^^1^ without cycloheximide; (3) 4 cycloheximide-only treatments (CY) at concentrations of 2, 4.5, 6, and 10 mg g^−^^1^ without streptomycin; and (4) 16 combined treatments (ST + CY) representing all possible concentration combinations of both inhibitors (2, 4.5, 6, and 10 mg g^−^^1^ for each inhibitor). This comprehensive design enabled a systematic evaluation of individual and interactive effects across the complete concentration gradient of both microbial inhibitors. The gas in the headspace of the flask was sampled, after 4 and 8 h of incubation, using a syringe to transfer the sample into a 12 mL headspace bottle (which had been evacuated). Gas samples were stored until analysis. After gas sampling, destructive sampling was performed on all the treated soils, and the leaching of soil inorganic N and soluble organic C was immediately conducted on the collected soil samples.

### 2.3. Measurements and Analysis

#### 2.3.1. Gas Sampling and Analysis for Sterilization Experiment

Following the treatment’s application in the sterilization experiment, all the flasks were sealed with bromobutyl rubber septa and aluminum caps (Macherey-Nagel, GmbH & Co. KG, Düren, Germany) and flushed with an O_2_-He mixture (21% O_2_, *v*/*v*) using five vacuum–replenishment cycles to minimize background gases. Headspace pressure was adjusted to atmospheric levels using a water-filled syringe. The flasks were incubated at 20 ± 0.5 °C in an automated incubation-monitoring system (Robot system) [24], which continuously measured N_2_O concentrations at 8 h intervals [25]. Gas sampling was performed using a peristaltic pump (Gilson Model 222, Gilson S.A.S., Villiers-le-Bel, France) connected to an Agilent 7890A gas chromatograph (Agilent Technologies Inc., Santa Clara, CA, USA). N_2_O fluxes were calculated from concentration changes between sampling intervals, following the NMBU methodology (Norwegian University of Life Sciences).

#### 2.3.2. Gas Sampling and Analysis for Biology Inhibitor Experiment

The N_2_O concentrations in the gas samples from the biology inhibitor experiment were analyzed using an Agilent 6890 gas chromatograph equipped with an electron capture detector (ECD) (Agilent Technologies Inc., Santa Clara, CA, USA). Soil gas fluxes were calculated using the following equation:

(1) $$F=\frac{\rho \;\times \;V\;\times \;\Delta c\;\times \;273}{\Delta t\;\times \;m\;\times \;\left(273+T\right)},$$

where F is the gas production rate (mg N g^−1^h^−1^); ρ is the gas density in the standard state—N_2_O is 1.964 kg^−1^ m^3^, CO_2_ is 1.997 kg^−1^ m^3^; V is the volume occupied by the gas in the culture bottle, which was 100 mL in this experiment; Δc is the gas concentration difference (ppmv); Δt is the time interval between two adjacent measurements (h); and T is the temperature at which the gas is measured, which in this experiment was 25 °C.

#### 2.3.3. Chemical and Physical Analysis

The soil was extracted using a 1 M KCl (Sinopharm Chemical Reagent Co., Ltd., Shanghai, China) solution at a soil/KCl solution ratio of 1:5 (*w*/*v*) immediately after incubation, and concentrations of NH_4_^+^-N and NO_3_^−^-N were measured using a continuous flow analyzer (AA3, Seal Analytical, Norderstedt, Germany).

DOC and DON were extracted with a 0.5 M K_2_SO_4_ solution and then measured by a TOC/TN instrument (TOC-VCSH, Shimadzu, Kyoto, Japan).

The calculation method of N conversion intensity (mg N kg^−1^ h^−1^) is as follows:

(2) $$\mathrm{Net}\;\mathrm{Nitrification}\;\mathrm{rate}=\frac{NO_{3^{-}}\text{-}N\;(\mathrm{AI})-{\mathrm{NO}}_{3^{-}}\text{-}N\;(\mathrm{BI})}{\mathrm{incubation}\;\mathrm{time}},$$

(3) $$\mathrm{Net}\;\mathrm{mineralization}\;\mathrm{rate}=\frac{[{{\mathrm{NH}}_{4}}^{+}\text{-}N\;(\mathrm{AI})+{\mathrm{NO}}_{3^{-}}\text{-}N\;(\mathrm{AI})]-[{{\mathrm{NH}}_{4}}^{+}\text{-}N\;(\mathrm{BI})+{\mathrm{NO}}_{3^{-}}\text{-}N\;(\mathrm{BI})]}{\mathrm{incubation}\;\mathrm{time}},$$

where (AI) = after incubation, (BI) = before incubation.

### 2.4. Statistical Analysis

Statistical analyses were performed using one-way ANOVA in SPSS 20.0 (IBM Corporation, Armonk, NY, USA), with post hoc LSD tests for multiple comparisons (*p* < 0.05). Data are presented as means ± standard error (SE) in all figures and tables, which were generated using SigmaPlot 15.0 (Systat Software Inc., San Jose, CA, USA) and Origin 2024 (OriginLab Corporation, Northampton, MA, USA) software.

## 3. Results

### 3.1. Effects of Biotic and Abiotic Interactions on Soil N_2_O Emission

**Figure 1:**
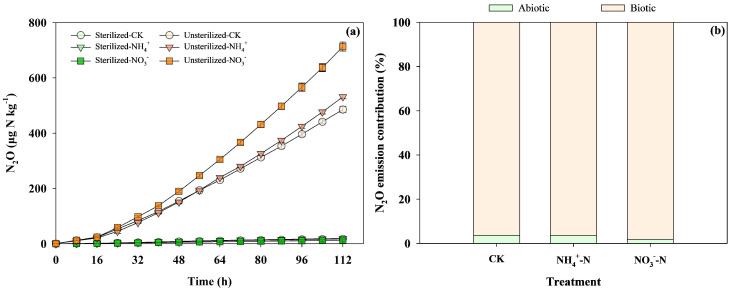
(**a**) Cumulative N_2_O emissions after 112 h of incubation in highly acidic soils under the application of different treatments, including treatments with the application of microbial inhibitors (1: Sterilized-CK, 2: Sterilized-NH_4_^+^-N, 3: Sterilized-NO_3_^−^-N) and treatments without applying microbial inhibitors (1: Sterilized-CK, 2: Sterilized-NH_4_^+^-N, 3: Sterilized-NO_3_^−^-N). (**b**) Effect of biotic and abiotic interaction on soil N_2_O emission in different treatments including: CK (control), NH_4_^+^-N (ammonium nitrogen), NO_3_^−^-N (nitrate nitrogen).

The experimental results revealed significant differences in N_2_O emissions between sterilized and unsterilized treatments, as illustrated in Figure 1. Specifically, unsterilized soils exhibited significantly higher N_2_O emissions, ranging from 486 to 713 μg N kg^−1^, compared to sterilized soils, which only produced 12.6 to 18.9 μg N kg^−1^. Within the unsterilized treatments, the NO_3_^−^-N amendment resulted in the highest cumulative N_2_O emissions (713 μg N kg^−1^), followed by NH_4_^+^-N (532 μg N kg^−1^) and the control (486 μg N kg^−1^). In sterilized treatments, N_2_O emissions remained minimal, with NH_4_^+^-N yielding 18.9 μg N kg^−1^, the control yielding 17.6 μg N kg^−1^, and NO_3_^−^-N yielding 12.6 μg N kg^−1^. Quantitative analysis indicated that biotic processes accounted for 96–98% of total N_2_O emissions, while abiotic contributions were negligible (1–3%). The NO_3_^−^-N treatment had the highest relative biotic contribution (98%), followed by the NH_4_^+^-N and control treatments (96% each).

### 3.2. Effects of Microbial Inhibitors on Soil N_2_O Flux

**Figure 2:**
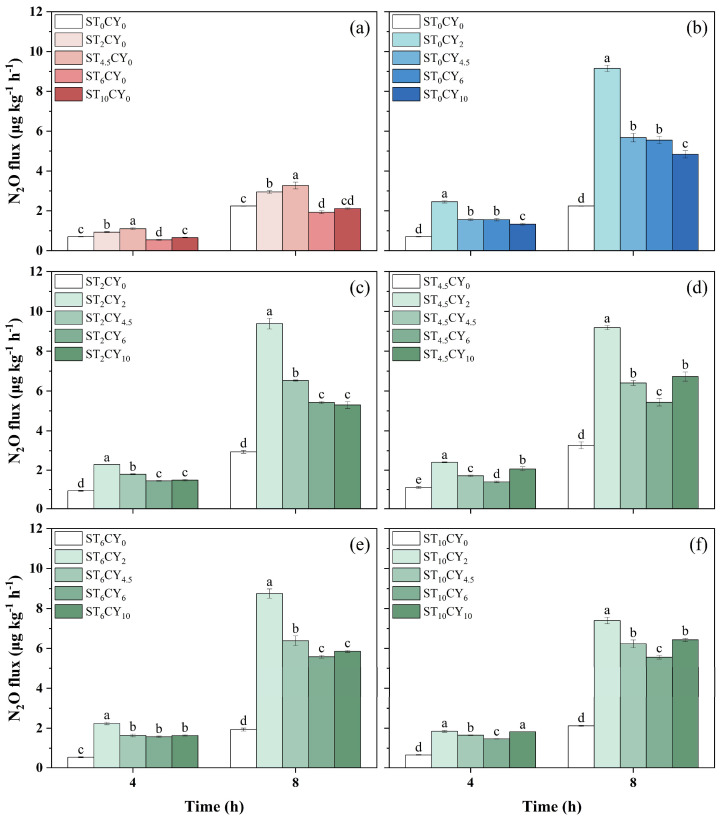
The N_2_O flux after 4 and 8 h of incubation in highly acidic soils under the application of microbial inhibitors (streptomycin and cycloheximide) with different concentrations, including (0, 2, 4.5, 6, 10 mg g^−1^) applied alone and with different combinations of both microbial inhibitors. Cycloheximide concentration was maintained at 0 mg g^−1^ (**a**) with varying streptomycin concentrations, while streptomycin concentrations were maintained at 0 (**b**), 2 (**c**), 4.5 (**d**), 6 (**e**), and 10 (**f**) mg g^−1^ with varying cycloheximide concentrations. Error bars represent standard errors, with different lowercase letters indicating statistically significant differences among treatments (*p* < 0.05).

The application of microbial inhibitors, streptomycin and cycloheximide, significantly influenced N_2_O emissions across treatments, as illustrated in Figure 2. N_2_O fluxes ranged from 0.541 to 2.45 μg kg^−1^ h^−1^ at 4 h and from 1.93 to 9.38 μg kg^−1^ h^−1^ at 8 h. In the absence of inhibitors, N_2_O emissions were 0.701 μg kg^−1^ h^−1^ at 4 h and 2.24 μg kg^−1^ h^−1^ at 8 h. High concentrations of streptomycin (6 and 10 mg g^−1^) reduced N_2_O emissions from 2.24 μg kg^−1^ h^−1^ to 1.93 μg kg^−1^ h^−1^ and 2.12 μg kg^−1^ h^−1^, respectively, whereas lower concentrations (2 and 4.5 mg g^−1^) increased emissions from 2.24 μg kg^−1^ h^−1^ to 2.95 μg kg^−1^ h^−1^ and 3.27 μg kg^−1^ h^−1^, respectively. The application rate of 6 mg g^−1^ showed the lowest emission of 1.93 μg kg^−1^ h^−1^ at 8 h among all the microbial inhibitor treatments, as the CK treatment showed emissions of 2.24 μg kg^−1^ h^−1^ at 8 h of incubation. Cycloheximide at 2 mg g^−1^ and 4.5 mg g^−1^ significantly enhanced N_2_O emissions at 8 h, reaching 9.15 μg kg^−1^ h^−1^ and 5.68 μg kg^−1^ h^−1^, respectively. However, higher dosages (6 mg g^−1^ and 10 mg g^−1^) inhibited N_2_O emissions compared to lower-dosage treatments, reducing them to 5.55 μg kg^−1^ h^−1^ and 4.84 μg kg^−1^ h^−1^, respectively. Furthermore, applying both inhibitors in combination showed significantly different results. The treatment ST_2_CY_2_ exhibited the highest N_2_O emissions of 9.38 μg kg^−1^ h^−1^. In contrast, treatments ST_2_CY_10_ and ST_4.5_CY_6_ showed inhibitory effects, with the lowest N_2_O emissions recorded at 5.30 μg kg^−1^ h^−1^ and 5.43 μg kg^−1^ h^−1^ at 8 h. The combined application of the two inhibitors significantly increased N_2_O emissions, with flux ranging from 1.39 to 9.38 μg kg^−1^ h^−1^. However, the combined application of the two inhibitors resulted in lower N_2_O emissions than the sum of their individual effects. For example, the ST_2_CY_2_ treatment produced 9.38 μg kg^−1^ h^−1^ N_2_O at 8 h, lower than the sum of individual ST_2_CY_0_ (2.95 μg kg^−1^ h^−1^) and ST_0_CY_2_ (9.15 μg kg^−1^ h^−1^) effects. The addition of cycloheximide to streptomycin treatment significantly increased N_2_O emissions, whereas the reverse combination had no such effect. For instance, the ST_2_CY_2_ treatment exhibited significantly higher N_2_O emissions than ST_2_CY_0_ at 8 h, while showing no significant difference from ST_0_CY_2_.

### 3.3. Effects of Microbial Inhibitors on Soil CO_2_ Flux

**Figure 3:**
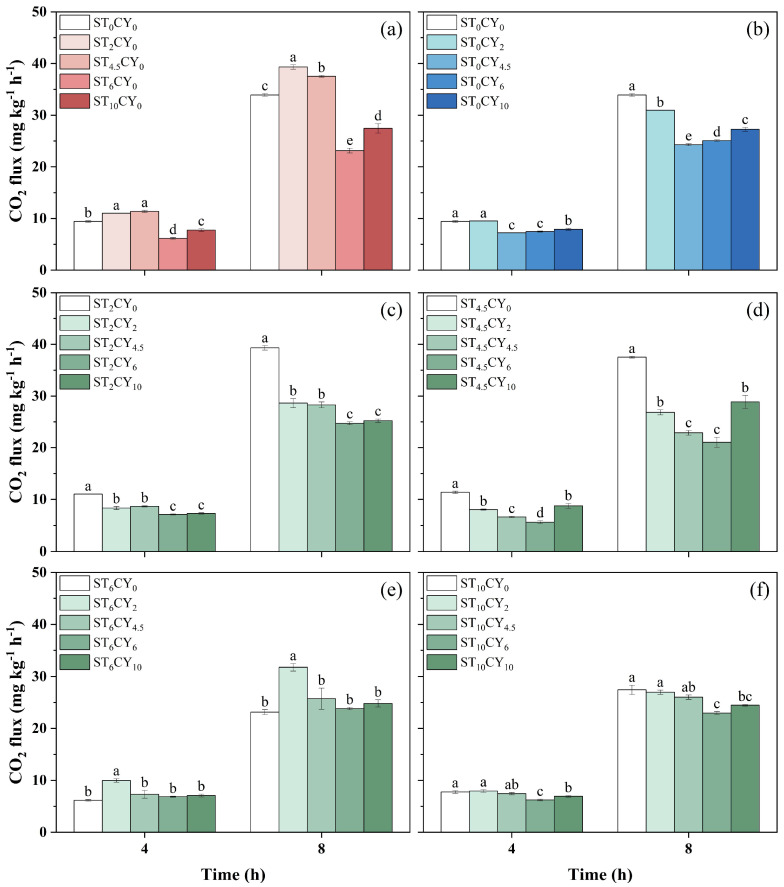
The CO_2_ flux after 4 and 8 h of incubation in highly acidic soils under the application of microbial inhibitors (streptomycin and cycloheximide) with different concentrations, including (0, 2, 4.5, 6, 10 mg g^−1^) applied alone and with different combinations of both microbial inhibitors. Cycloheximide concentration was maintained at 0 mg g^−1^ (**a**) with varying streptomycin concentrations, while streptomycin concentrations were maintained at 0 (**b**), 2 (**c**), 4.5 (**d**), 6 (**e**), and 10 (**f**) mg g^−1^ with varying cycloheximide concentrations. Error bars represent standard errors, with different lowercase letters indicating statistically significant differences among treatments (*p* < 0.05).

The application of microbial inhibitors, streptomycin and cycloheximide, significantly influenced CO_2_ emissions across different treatments, as shown in Figure 3. The CO_2_ fluxes ranged from 5.62 to 11.4 mg kg^−1^ h^−1^ at 4 h and from 21.1 to 39.3 mg kg^−1^ h^−1^ at 8 h. For soils without the addition of an inhibitor, CO_2_ emission was 9.42 mg kg^−1^ h^−1^ and 33.9 mg kg^−1^ h^−1^ at 4 and 8 h, respectively. In general, CO_2_ emission decreased with inhibitor dosages. However, when treated with 2 mg g^−1^ and 4.5 mg g^−1^ streptomycin, the CO_2_ emissions increased significantly, reaching 39.3 mg kg^−1^ h^−1^ and 37.5 mg kg^−1^ h^−1^ at 8 h, respectively. The combined application of the two inhibitors significantly decreased CO_2_ emissions (ranging from 5.62 to 31.7 mg kg^−1^ h^−1^), except ST_6_CY_2_ at 4 h. The combined application of the two inhibitors resulted in lower CO_2_ emissions than the sum of their individual effects. For example, the ST_6_CY_2_ treatment produced 31.7 mg kg^−1^ h^−1^ at 8 h, lower than the sum of individual ST_6_CY_0_ (23.1 mg kg^−1^ h^−1^) and ST_0_CY_2_ (30.9 mg kg^−1^ h^−1^) effects.

### 3.4. Effects of Microbial Inhibitors on Soil Available Nitrogen and Carbon Contents

The NH_4_^+^-N and NO_3_^−^-N content in soils was affected by the application of microbial inhibitors, as shown in Figures S1 and S2. Without inhibitors, NH_4_^+^-N content was 11.0 mg kg^−1^. Streptomycin increased the NH_4_^+^-N to 15.2–21.7 mg kg^−1^, while cycloheximide elevated it to 24.6–25.3 mg kg^−1^. Among different streptomycin dosages without cycloheximide, the NH_4_^+^-N content was significantly higher with 10 mg g^−1^ than that with lower dosages. NO_3_^−^-N content without inhibitors was 181 mg kg^−1^, varying between 170 and 186 mg kg^−1^ across treatments. The treatments involving the application of 10 mg g^−1^ of streptomycin combined with cycloheximide, as well as the ST_6_CY_6_ and ST_6_CY_10_ treatments, significantly reduced the NO_3_^−^-N content.

**Figure 4:**
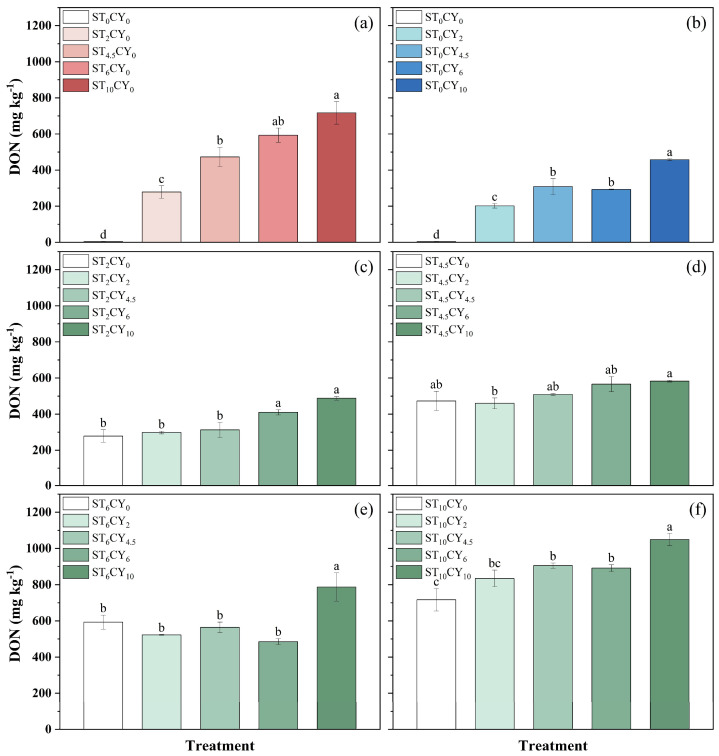
Influence on the concentration of dissolved organic nitrogen (DON) after incubation in highly acidic soils of the application of microbial inhibitors (streptomycin and cycloheximide) with different concentrations, including (0, 2, 4.5, 6, 10 mg g^−1^) applied alone and with different combinations of both microbial inhibitors. Cycloheximide concentration was maintained at 0 mg g^−1^ (**a**) with varying streptomycin concentrations, while streptomycin concentrations were maintained at 0 (**b**), 2 (**c**), 4.5 (**d**), 6 (**e**), and 10 (**f**) mg g^−1^ with varying cycloheximide concentrations. Error bars represent standard errors, with different lowercase letters indicating statistically significant differences among treatments (*p* < 0.05).

**Figure 5:**
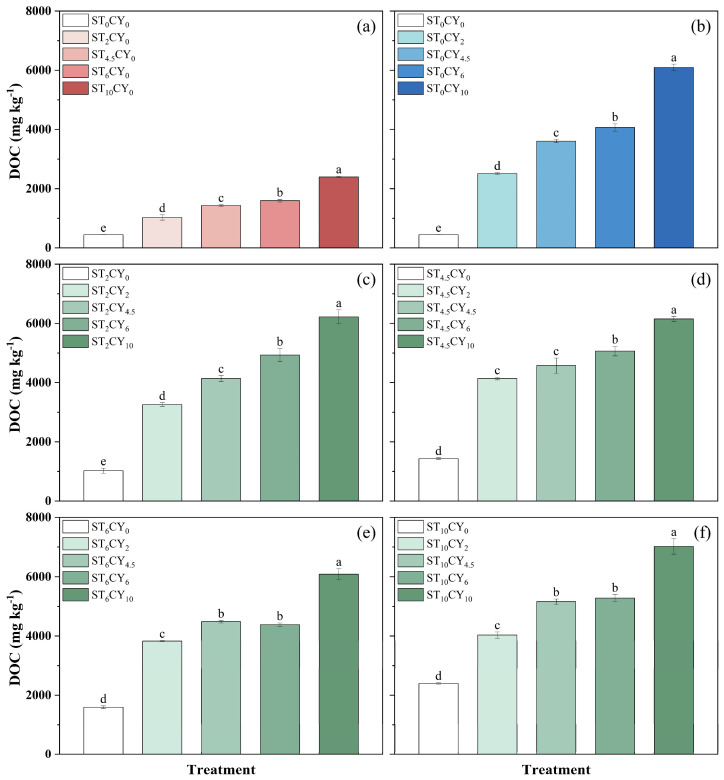
Influence on the concentration of dissolved organic carbon (DOC) after incubation in highly acidic soils of the application of microbial inhibitors (streptomycin and cycloheximide) with different concentrations, including (0, 2, 4.5, 6, 10 mg g^−1^) applied alone and with different combinations of both microbial inhibitors. Cycloheximide concentration was maintained at 0 mg g^−1^ (**a**) with varying streptomycin concentrations, while streptomycin concentrations were maintained at 0 (**b**), 2 (**c**), 4.5 (**d**), 6 (**e**), and 10 (**f**) mg g^−1^ with varying cycloheximide concentrations. Error bars represent standard errors, with different lowercase letters indicating statistically significant differences among treatments (*p* < 0.05).

The soil DON and DOC content was significantly influenced by the application of microbial inhibitors, as shown in Figure 4 and Figure 5. Soil DON content in the soils without inhibitors was 3.59 mg kg^−1^, increasing to 278–717 mg kg^−1^ with streptomycin and 202–457 mg kg^−1^ with cycloheximide treatment, showing an overall upward trend with an increasing inhibitor dosage. The same was true when the two inhibitors were applied in combination, with DON content ranging from 298 to 1049 mg kg^−1^. The combined application of the two inhibitors resulted in a lower DON content than the sum of their individual effects. For example, the DON concentration in treatment ST_2_CY_2_ was 298 mg kg^−1^, lower than the sum of individual ST_2_CY_0_ (278 mg kg^−1^) and ST_0_CY_2_ (202 mg kg^−1^) effects. Under the same addition levels, the DON content in samples treated with streptomycin was higher than that in samples treated with cycloheximide. The highest DON content was observed in the combined treatment of 10 mg g^−^^1^ streptomycin and 10 mg g^−^^1^ cycloheximide (ST_10_CY_10_). The DOC content in the soils without inhibitors’ addition was 444 mg kg^−1^. The DOC content significantly increased with inhibitors’ addition. The DOC content of different treatments varied from 444 mg kg^−1^ to 7018 mg kg^−1^. Under the same addition levels, the DOC content in samples treated with streptomycin was lower than that in samples treated with cycloheximide. Both streptomycin and cycloheximide individually enhanced DOC accumulation in a dose-dependent manner, with higher concentrations resulting in greater increases. And the combination of the two inhibitors further amplified this effect. The highest DOC content was shown in soil treated with 10 mg g^−1^ streptomycin combined with 10 mg g^−1^ cycloheximide, with values of 7018 mg kg^−1^. The combined application of the two inhibitors resulted in lower DOC content than the sum of their individual effects, except ST_4.5_CY_2_. For example, the DOC concentration in treatment ST_2_CY_2_ was 3258 mg kg^−1^, lower than the sum of individual ST_2_CY_0_ (1024 mg kg^−1^) and ST_0_CY_2_ (2511 mg kg^−1^) effects.

### 3.5. Correlation of N_2_O and Soil Properties with Microbial Inhibitor Dosages

**Figure 6:**
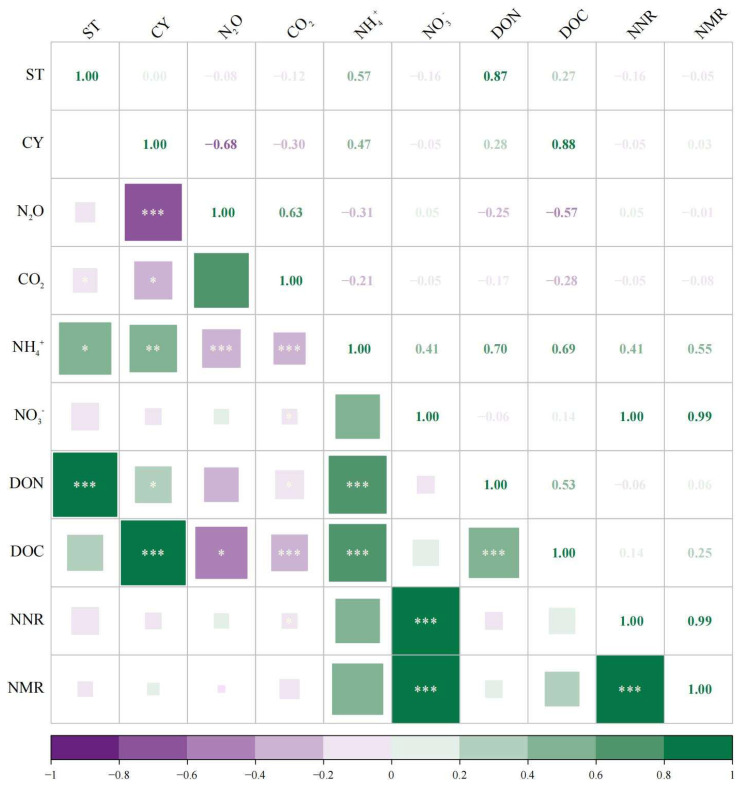
Correlation heatmap of gaseous and soil properties with microbial inhibitor dosages. The size of each square is proportional to the absolute value of the correlation coefficient. ST: streptomycin dosage; CY: cycloheximide dosage; NNR: net nitrification rate; NMR: net mineralization rate. *, *p* < 0.05; **, *p* < 0.01; ***, *p* < 0.001.

N_2_O emissions and soil properties were significantly influenced by microbial inhibitors, as shown in Figure 6. The Pearson correlation analysis indicated that N_2_O emission was negatively correlated with cycloheximide dosage (R = −0.68, *p* < 0.001), and it was also negatively associated with NH_4_^+^-N (R = −0.31, *p* < 0.001) and DOC (R = −0.57, *p* < 0.05). In terms of soil properties, soil’s NH_4_^+^-N content was significantly positively correlated with streptomycin (R = 0.57, *p* < 0.05) and cycloheximide (R = 0.47, *p* < 0.01) dosage. Soil’s DON content was significantly positively correlated with streptomycin (R = 0.87, *p* < 0.001) and cycloheximide (R = 0.28, *p* < 0.05) dosage. Soil’s DOC content was significantly positively correlated with cycloheximide (R = 0.88, *p* < 0.001) dosage.

## 4. Discussion

### 4.1. Effects of Microbial Inhibitors on N_2_O Production Pathways

N_2_O is primarily produced through microbially driven N cycling processes [26]. These processes can be significantly influenced by microbial inhibitors, which selectively target specific microbial groups and alter their activity [27]. In strongly acidic vegetable soils, fungal contributions to N_2_O production are particularly pronounced due to their greater tolerance to acidic conditions compared to many bacteria [14]. The inhibition of microbial activity is typically expected to reduce N_2_O emission by suppressing key microbial groups involved in nitrification and denitrification [28,29]. For instance, streptomycin, a prokaryotic inhibitor, is expected to suppress ammonia-oxidizing bacteria (AOB) and nitrite-oxidizing bacteria (NOB), thereby reducing N_2_O production through nitrification [8]. Similarly, cycloheximide, a eukaryotic inhibitor, is expected to inhibit fungal activity, thereby reducing heterotrophic nitrification [30] and fungal denitrification [31]. However, the findings of this study revealed a more complex pattern of responses to microbial inhibitors.

High concentrations of streptomycin (6 mg g^−1^ and 10 mg g^−1^) resulted in a decrease in N_2_O emissions (Figure 2). This finding aligns with previous studies demonstrating that streptomycin can effectively suppress bacterial activity, leading to decreased N_2_O production [18]. However, the N_2_O generation rate of most soil samples treated with this inhibitor did not decrease but increased. This pattern was consistent across individual applications of streptomycin and cycloheximide, as well as their combined treatments. Previous research indicated that microbial inhibition could trigger compensatory responses from surviving microorganisms [32,33]. The decomposition of microbial necromass can provide additional substrates for N_2_O production pathways such as nitrification and denitrification [34]. Additionally, microbial inhibitors themselves can serve as sources of C and N, further fueling microbial activity and the generation of N_2_O [20,35]. The increase in N_2_O production can thus be attributed to a balance between microbial death-induced substrate release and compensatory microbial responses [36,37]. The results of this study indicate that the effects of microbial inhibitors on N_2_O emission are not straightforward and can vary depending on the concentration and type of inhibitor used. This complexity arises from the intricate interplay between microbial community structure, functional redundancy, and the availability of substrates for the production of N_2_O.

The observed effects of microbial inhibitors on N_2_O emission in strongly acidic vegetable soils highlight the importance of considering the unique characteristics of these environments. Acidic soils are known to harbor distinct microbial communities with specialized adaptations to low pH conditions, including acidophilic taxa [13]. These acidophilic microorganisms can maintain active nitrification and denitrification processes even at pH levels below 4.5, contributing significantly to N_2_O emission [12]. Therefore, microbial imbalance in acidic soils can have unintended consequences, such as shifts in the microbial community structure and enhanced N_2_O production through compensatory mechanisms. It also highlights the importance of considering the broader ecological context in which these inhibitors are applied. Agricultural practices, such as the use of fertilizers and organic amendments, can significantly influence soil’s microbial communities and their associated biogeochemical processes. Understanding these complex interactions is crucial for developing sustainable strategies to reduce N_2_O emissions while maintaining soil fertility and ecosystems’ functionality.

### 4.2. Effects of Microbial Inhibitors on the Dynamics of Dissolved Nitrogen and Carbon Fractions

NH_4_^+^-N and NO_3_^−^-N serve as substrates for N_2_O production [38], and microbial inhibitors can influence N_2_O emissions by affecting the microbial utilization of these nitrogen substrates. The significant increase in NH_4_^+^-N content following the application of microbial inhibitors suggests that the inhibition of microbial N transformation (as shown in Figures S3 and S4) processes, such as nitrification, leads to the accumulation of NH_4_^+^-N in soil. This is likely due to the reduced activity of ammonia-oxidizing bacteria and archaea, which are key players in the nitrification pathway [39]. The inhibitors impede protein synthesis in certain fungi and bacteria, thereby reducing their biological activities, slowing nitrification, and decreasing NH_4_^+^-N consumption. Additionally, archaea contribute significantly to nitrification in acidic soil [40,41]. Cycloheximide may inhibit ammonia-oxidizing archaea [42], reducing the nitrification rate and further contributing to the accumulation of NH_4_^+^-N.

The concurrent increase in the soil’s DON and DOC indicates that microbial inhibitors not only affect N cycling but also alter the availability of organic substrates (Figure 4 and Figure 5). The decomposition of microbial biomass and the release of intracellular compounds likely contribute to these elevated levels, influencing microbial respiration and C mineralization rates [21]. Furthermore, the death of some microorganisms due to the application of an inhibitor creates a biological pulse, providing a substrate for surviving microorganisms [43]. Lower concentrations of cycloheximide may cause fungal cell lysis [44], supplying C and N substrates to the remaining microbial community, which enhances NH_4_^+^-N availability, microbial respiration, and mineralization rates [33], consistent with the enhanced CO_2_ production and mineralization rates observed in our experiments for soil samples treated with inhibitors.

The observed increase in soil’s DON and DOC content following the application of microbial inhibitors suggests that the decomposition of microbial biomass and release of intracellular compounds contribute to these elevated levels. This has significant implications for microbial respiration, C mineralization rates, and the dynamics of soil organic matter [45]. Increased DOC content can enhance microbial activity and respiration, leading to elevated CO_2_ emissions and potential changes in soil C storage [46]. This highlights the importance of considering the broader impacts of microbial inhibitors on soil C cycling and the potential feedbacks to climate change.

In this study, we conducted experiments in strongly acidic vegetable soils. While our findings offer valuable insights into N_2_O emissions under microbial suppression conditions in such highly acidic soils, the results may not be directly applicable to other soil types or management practices. A meta-analysis has demonstrated that the impact of inhibitors on N_2_O emissions, NH_4_^+^-N, ammonification, nitrate, and nitrification is contingent upon soil type [21]. Specifically, fungal inhibitors have been shown to mitigate N_2_O emissions in agricultural soils, to significantly diminish N_2_O emissions in grassland soils, and to augment N_2_O emissions in forest soils [21]. Fungal communities display a higher degree of sensitivity to tillage and cover cropping practices compared to bacterial communities [47]. Consequently, when inhibitors are applied, distinct agricultural management practices can elicit divergent effects on soil N_2_O emissions.

## 5. Conclusions

This study concludes that microbial communities’ imbalance, induced by selective inhibition in strongly acidic vegetable soils, has significant implications for N_2_O emissions and N dynamics. High concentrations of microbial inhibitors can effectively reduce N_2_O emission, likely by suppressing key microbial groups involved in nitrification and denitrification. However, lower concentrations may trigger compensatory responses from surviving microorganisms, leading to increased N_2_O production. The observed increases in DOC and DON levels suggest that microbial inhibitors can stimulate microbial decomposition, providing additional substrates for N_2_O production and potentially exacerbating community imbalances. These findings underscore the complexity of microbial interactions in acidic soils and highlight the importance of considering the broader ecological context when applying microbial inhibitors. Future research should focus on long-term ecological impacts and explore sustainable management strategies to mitigate N_2_O emissions while preserving soil’s health and agricultural productivity.

## Data Availability

Data will be made available on request.

## Supplementary Materials

The following supporting information can be downloaded at: https://www.mdpi.com/article/10.3390/biology14060621/s1, Figure S1: Influence on the concentration of ammonium nitrogen (NH_4_^+^-N) after incubation in highly acidic soils of the application of microbial inhibitors (streptomycin and cycloheximide) with different concentrations, including (0, 2, 4.5, 6, 10 mg g^−1^) applied alone and with different combinations of both microbial inhibitors. Figure S2: Influence on the concentration of nitrate nitrogen (NO_3_^−^-N) after incubation in highly acidic soils of the application of microbial inhibitors (cycloheximide and streptomycin) with different concentrations, including (0, 2, 4.5, 6, 10 mg g^−1^) applied alone and with different combinations of both microbial inhibitors. Figure S3: Influence on the concentration of net nitrification rate (NNR) after incubation in highly acidic soils of the application of microbial inhibitors (streptomycin and cycloheximide) with different concentrations, including (0, 2, 4.5, 6, 10 mg g^−1^) applied alone and with different combinations of both microbial inhibitors. Figure S4: Influence on the concentration of net mineralization rate (NMR) after incubation in highly acidic soils of the application of microbial inhibitors (streptomycin and cycloheximide) with different concentrations, including (0, 2, 4.5, 6, 10 mg g^−1^) applied alone and with different combinations of both microbial inhibitors.
