# Supplementary material for: Microbial Community Imbalance Drives Nitrous Oxide Emissions from Strongly Acidic Soil—Insights from a Laboratory Experiment with Microbial Inhibitors
Source: Biology (Basel). 2025 May 28;14(6):621. doi: 10.3390/biology14060621 (PMC12189618; doi:10.3390/biology14060621)
Supplement: Supplementary file 1 [file biology-14-00621-s001.zip › biology-3615105-supplementary.pdf]

Supplementary information

Microbial Community Imbalance Drives Nitrous Oxide Emissions from Strongly Acidic Soil—Insights from a Laboratory Experiment with Microbial Inhibitors

Waqar Ahmed [1,2,†], Hongyang Gong [1,2,†], Xiaoxiao Xiang [2], Runze Chen [2], Yumeng Xu [2], Wenxuan Shi [3], Binzhe Li [4], Junhui Yin [1,*] and Qing Chen [2]

1 State Key Lab of Biocontrol, Guangdong Provincial Key Laboratory of Plant Stress Biology, School of Agriculture and Biotechnology, Shenzhen Campus of Sun Yat-sen University, Shenzhen 518107, China; waqarahmedupr@gmail.com (W.A.); hygong@cau.edu.cn (H.G.)

2 College of Resources and Environmental Sciences, China Agricultural University, Beijing 100193, China; xiangxx@cau.edu.cn (X.X.); crz15751136686@163.com (R.C.); 13470700223@163.com (Y.X.); qchen@cau.edu.cn (Q.C.)

3 Teagasc, Environmental Research Centre, Johnstown Castle, Co. Wexford, Y35 TC97, Ireland; wenxuan.shi@teagasc.ie

4 College of Engineering (Key Laboratory for Clean Renewable Energy Utilization Technology, Ministry of Agriculture), China Agricultural University, Beijing 100083, China; libinzhe@cau.edu.cn

* Correspondence: yinjh9@mail.sysu.edu.cn

† These authors contributed equally to this work.

[*]Correspondence: Junhui Yin, State Key Lab of Biocontrol, Guangdong Provincial Key Laboratory of Plant Stress Biology, School of Agriculture and Biotechnology, Shenzhen Campus of Sun Yat-sen University, Shenzhen, 518107, China. Email: yinjh9@mail.sysu.edu.cn

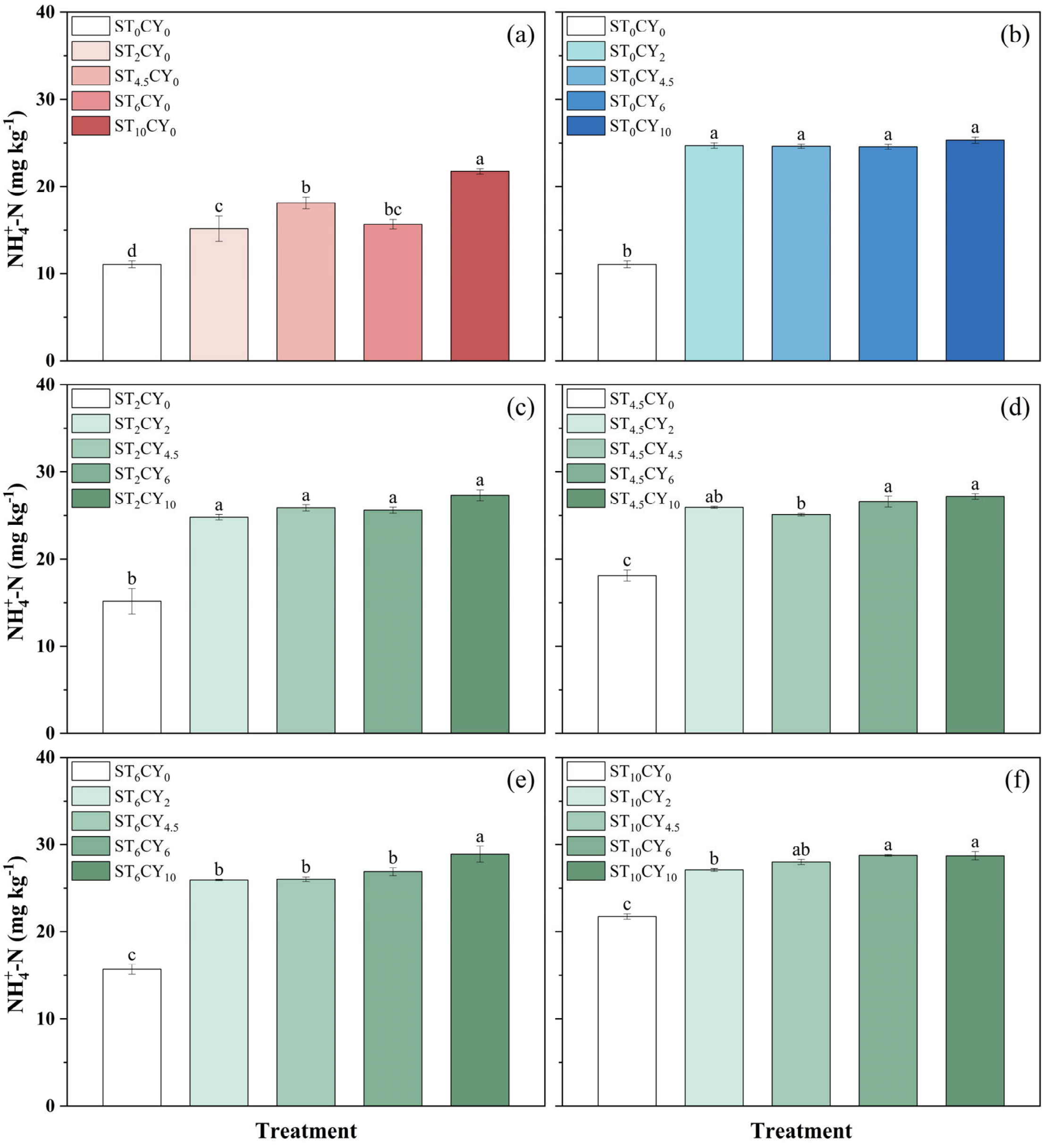

Figure S1. The Influence on the concentration of ammonium nitrogen (NH_4^+-N) after incubation in highly acidic soils under the application of microbial inhibitors (streptomycin and cycloheximide) with different concentrations including (0, 2, 4.5, 6, 10 mg g^{-1}) applied alone and with the different combinations of both microbial inhibitors. Cycloheximide concentration was maintained at 0 mg g^{-1} (a) with varying streptomycin concentrations, while streptomycin concentrations were maintained at 0 (b), 2 (c), 4.5 (d), 6 (e), and 10 (f) mg g^{-1} with varying cycloheximide concentrations.

Error bars represent standard errors, with different lowercase letters indicating statistically significant differences among treatments ($p < 0.05$).

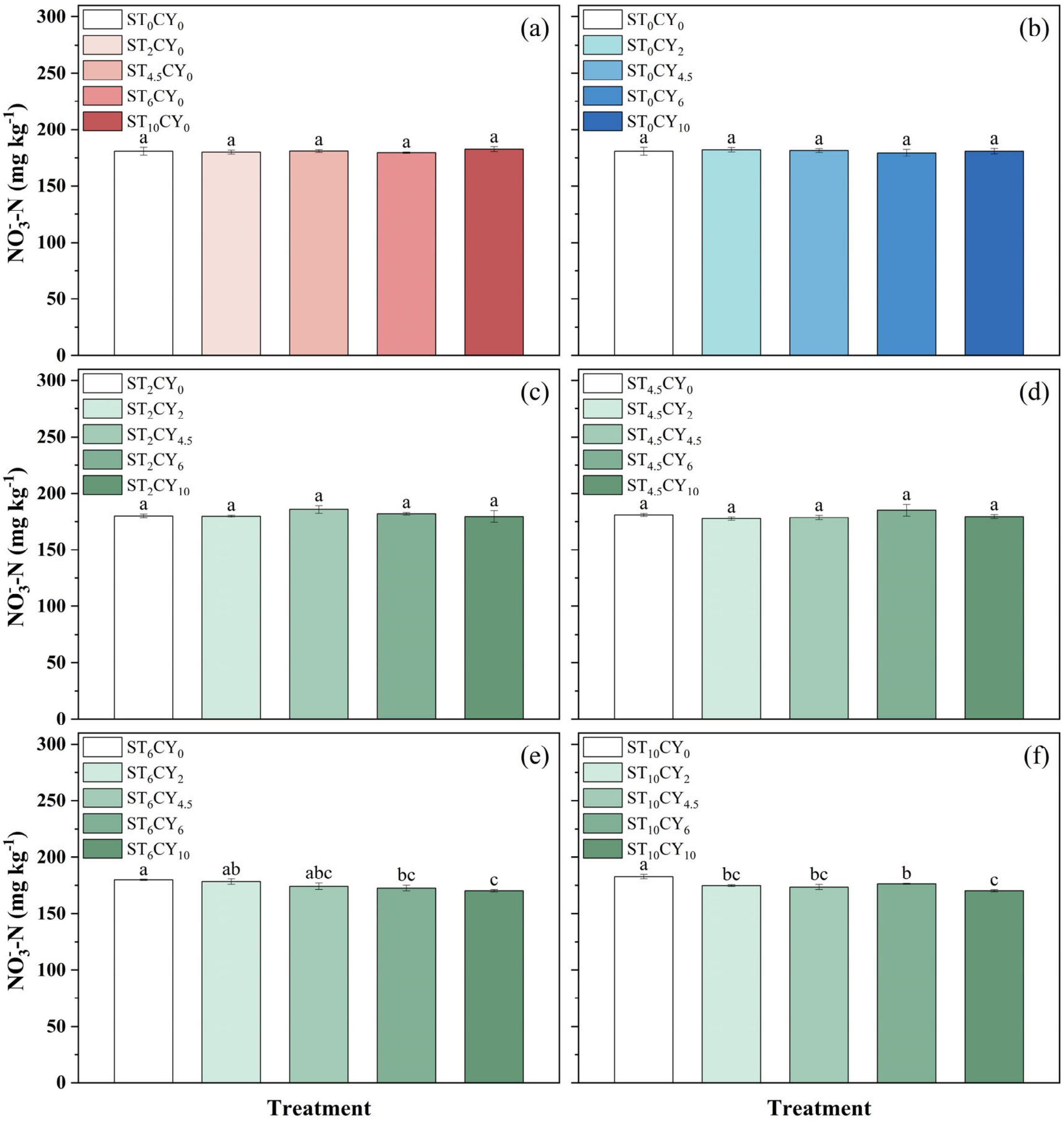

Figure S2. The Influence on the concentration of nitrate nitrogen (NO_3^--N) after incubation in highly acidic soils under the application of microbial inhibitors (cycloheximide and streptomycin) with different concentrations including (0, 2, 4.5, 6, 10 mg g^{-1}) applied alone and with the different combinations of both microbial inhibitors. Cycloheximide concentration was maintained at 0 mg g^{-1} (a) with varying streptomycin concentrations, while streptomycin concentrations were maintained at 0

(b), 2 (c), 4.5 (d), 6 (e), and 10 (f) mg g^{-1} with varying cycloheximide concentrations. Error bars represent standard errors, with different lowercase letters indicating statistically significant differences among treatments ($p < 0.05$).

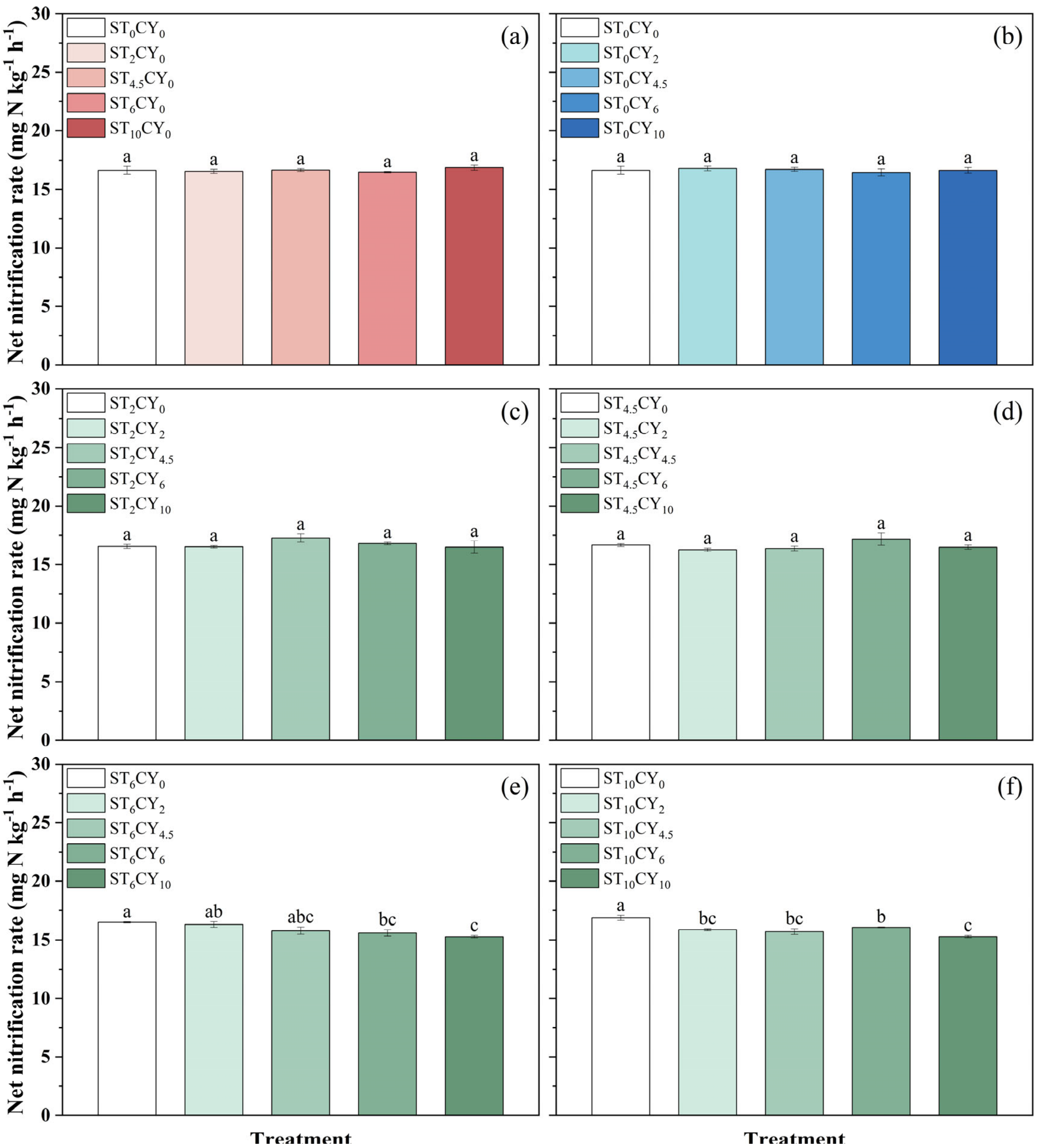

Figure S3. The Influence on the concentration of net nitrification rate (NNR) after incubation in highly acidic soils under the application of microbial inhibitors (streptomycin and cycloheximide) with different concentrations including (0, 2, 4.5, 6, 10 mg g^{-1}) applied alone and with the different combinations of both microbial

inhibitors. Cycloheximide concentration was maintained at 0 mg g^{-1} (a) with varying streptomycin concentrations, while streptomycin concentrations were maintained at 0 (b), 2 (c), 4.5 (d), 6 (e), and 10 (f) mg g^{-1} with varying cycloheximide concentrations. Error bars represent standard errors, with different lowercase letters indicating statistically significant differences among treatments ($p < 0.05$).

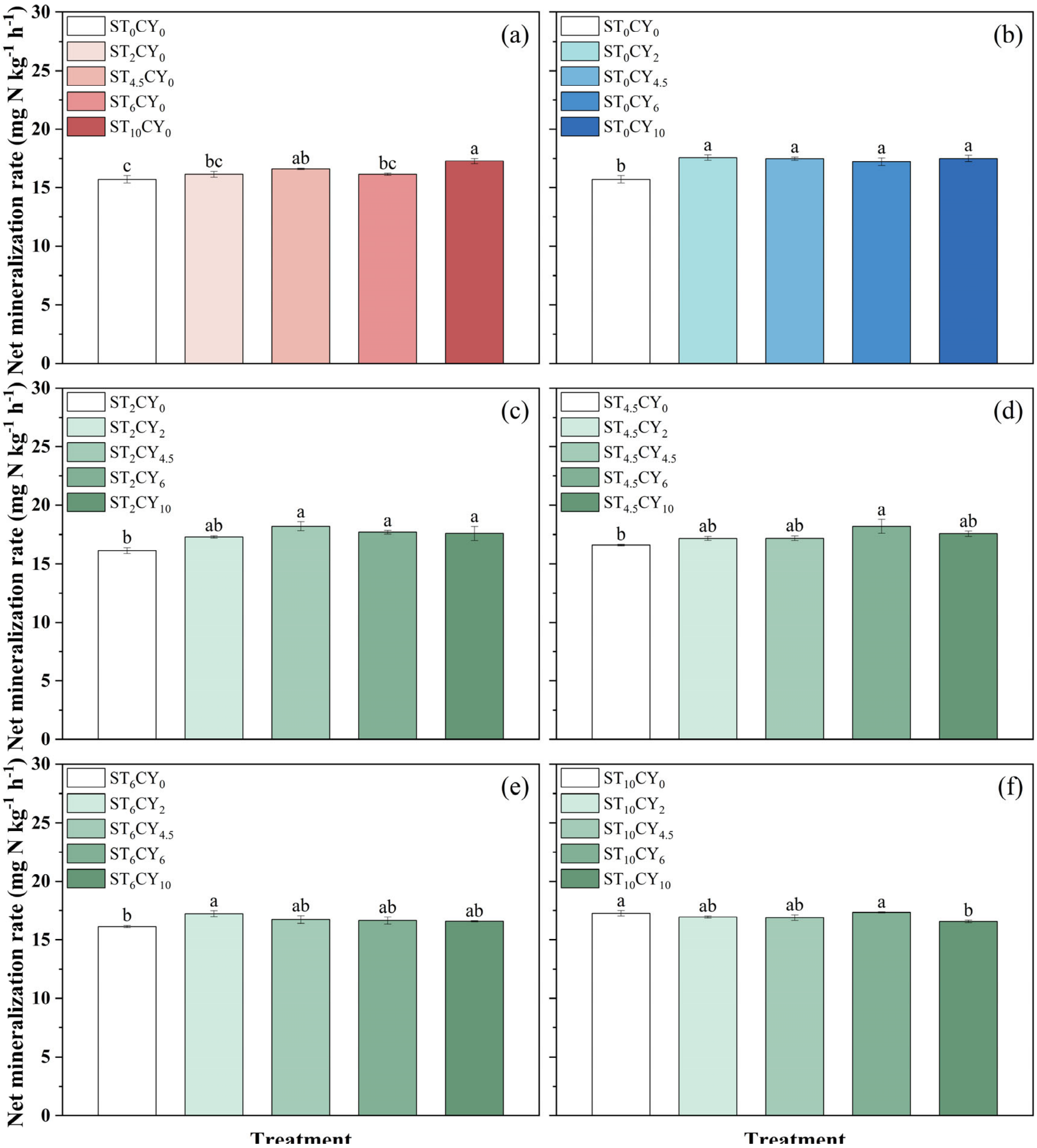

Figure S4. The Influence on the concentration of net mineralization rate (NMR) after incubation in highly acidic soils under the application of microbial inhibitors

(streptomycin and cycloheximide) with different concentrations including (0, 2, 4.5, 6, 10 mg g^{-1}) applied alone and with the different combinations of both microbial inhibitors. Cycloheximide concentration was maintained at 0 mg g^{-1} (a) with varying streptomycin concentrations, while streptomycin concentrations were maintained at 0 (b), 2 (c), 4.5 (d), 6 (e), and 10 (f) mg g^{-1} with varying cycloheximide concentrations. Error bars represent standard errors, with different lowercase letters indicating statistically significant differences among treatments ($p < 0.05$).
